# Efficient Cholesterol Transport in Dendritic Cells Defines Optimal Exogenous Antigen Presentation and Toxoplasma gondii Proliferation

**DOI:** 10.3389/fcell.2022.837574

**Published:** 2022-03-04

**Authors:** Cristina Croce, Facundo Garrido, Sofía Dinamarca, Julien Santi-Rocca, Sabrina Marion, Nicolas Blanchard, Luis S. Mayorga, Ignacio Cebrian

**Affiliations:** ^1^ Instituto de Histología y Embriología de Mendoza (IHEM) - Universidad Nacional de Cuyo - CONICET, Mendoza, Argentina; ^2^ Institut Toulousain des Maladies Infectieuses et Inflammatoires (Infinity), Inserm/CNRS/Université Toulouse 3, Toulouse, France; ^3^ CNRS, Inserm, CHU Lille, U1019 - UMR 9017 - CIIL - Center for Infection and Immunity of Lille, Institut Pasteur de Lille, Université de Lille, Lille, France; ^4^ Facultad de Ciencias Exactas y Naturales, Universidad Nacional de Cuyo, Mendoza, Argentina

**Keywords:** dendritic cells, cholesterol, intraluminal vesicles, multivesicular bodies, CHMP4b, *Toxoplasma gondii*, antigen presentation, U18666A inhibitor

## Abstract

Dendritic cells are the most powerful antigen-presenting cells of the immune system. They present exogenous antigens associated with Major Histocompatibility Complex (MHC) Class II molecules through the classical pathway to stimulate CD4+ T cells, or with MHC-I to activate CD8+ T lymphocytes through the cross-presentation pathway. DCs represent one of the main cellular targets during infection by *Toxoplasma gondii*. This intracellular parasite incorporates essential nutrients, such as cholesterol, to grow and proliferate inside a highly specialized organelle, the parasitophorous vacuole (PV). While doing so, *T. gondii* modulates the host immune response through multiple interactions with proteins and lipids. Cholesterol is an important cellular component that regulates cellular physiology at the structural and functional levels. Although different studies describe the relevance of cholesterol transport for exogenous antigen presentation, the molecular mechanism underlying this process is not defined. Here, we focus our study on the inhibitor U18666A, a drug widely used to arrest multivesicular bodies biogenesis that interrupts cholesterol trafficking and changes the lipid composition of intracellular membranes. Upon bone marrow-derived DC (BMDC) treatment with U18666A, we evidenced a drastic disruption in the ability to present exogenous soluble and particulate antigens to CD4+ and CD8+ T cells. Strikingly, the presentation of *T. gondii*-associated antigens and parasite proliferation were hampered in treated cells. However, neither antigen uptake nor BMDC viability was significantly affected by the U18666A treatment. By contrast, this drug altered the transport of MHC-I and MHC-II molecules to the plasma membrane. Since U18666A impairs the formation of MVBs, we analyzed in *T. gondii* infected BMDCs the ESCRT machinery responsible for the generation of intraluminal vesicles. We observed that different MVBs markers, including ESCRT proteins, were recruited to the PV. Surprisingly, the main ESCRT-III component CHMP4b was massively recruited to the PV, and its expression level was upregulated upon BMDC infection by *T. gondii*. Finally, we demonstrated that BMDC treatment with U18666A interrupted cholesterol delivery and CHMP4b recruitment to the PV, which interfered with an efficient parasite replication. Altogether, our results highlight the importance of cholesterol trafficking and MVBs formation in DCs for optimal antigen presentation and *T. gondii* proliferation.

## Introduction

Dendritic cells (DCs) are considered the most potent antigen presenting cells of the immune system. They present exogenous antigens on Major Histocompatibility Complex (MHC) class II molecules through the classical pathway to stimulate CD4+ T lymphocytes, or on MHC-I to activate CD8+ T cells through the cross-presentation pathway (Nutt and Chopin, 2020). On the one hand, newly synthesized MHC-II molecules associate with the invariant chain (Ii) in the ER, stabilizing the complex and preventing premature peptide binding. The cytoplasmic domain of Ii allows the translocation of the MHC-II/Ii complex from the Golgi to the endocytic network (Jurewicz and Stern, 2019). In this way, it reaches the MIIC compartment, where the requested proteins for efficient peptide loading are present (Rocha and Neefjes, 2008). MIIC compartments exhibit late endosomes characteristics (CD63+ and Lamp1+; Calafat et al., 1994), and there Ii is partially degraded, leaving a residual peptide attached to the peptide-binding site (CLIP) (Romagnoli and Germain, 1994). Moreover, MIIC compartments associate to other vesicles of the endocytic system, promoting the encounter with peptides derived from internalized antigens. The MIIC resident chaperone HLA-DM mediates the exchange of CLIP for the antigenic peptide (Jurewicz and Stern, 2019). Finally, MHC-II/peptide complexes reach the plasma membrane localizing at cholesterol-enriched domains (Bosch et al., 2013b).

On the other hand, during cross-presentation exogenous antigens uptaken by DCs are first partially degraded in the endocytic network, and then translocated to the cytosol, where they are further degraded by the proteasome (Kotsias et al., 2019). The generated peptides enter cross-presenting compartments to meet and be loaded onto MHC-I molecules. The intracellular source of MHC-I molecules for cross-presentation is still matter of debate, but different studies highlight the importance of the MHC-I pool present in the endocytic recycling compartment (ERC) (Nair-Gupta et al., 2014; Cebrian et al., 2016). There is also evidence about the interaction between MHC-I and Ii that impacts on cross-presentation, which is suggested to mediate the arrival of newly synthesized MHC-I to the endocytic network without affecting recycling (Basha et al., 2012). The specific location and intracellular compartment required for MHC-I/peptide complex formation is not fully defined (Blander, 2018), and this represents a key question in the field.

Cholesterol is an important component of biological membranes, being crucial both for maintaining their structure and for signaling events. Most of the cellular cholesterol is found in microdomains of the plasma membrane, called lipid rafts (Fessler, 2015). Lipid rafts serve as platforms where certain receptors and membrane proteins, such as MHC molecules, are grouped. An important role for lipid rafts grouping MHC-II/peptide complexes in order to activate immune responses was shown (Bosch et al., 2013b). Moreover, there is a cholesterol-binding site in the transmembrane domain of MHC-II, which allosterically modulates the loading of antigenic peptides, and contributes to the stabilization of MHC-II/peptide complexes at the plasma membrane (Roy et al., 2013). However, the role of cholesterol trafficking during MHC-II antigen presentation is not clearly defined yet. Regarding cross-presentation, it was reported that cationic lipids contribute to regulate antigen degradation by increasing the phagosomal pH (Gao et al., 2017). Furthermore, cholesterol depletion with lovastatin reduces macropinocytosis, and affects the cross-presentation ability of DCs (Albrecht et al., 2006). Increase in lipid bodies in IFNγ-activated DCs was found to correlate with improved cross-presentation efficacy (Bougnères et al., 2009; den Brok et al., 2016), although the underlying mechanism has not been identified.

*Toxoplasma gondii* is an obligate intracellular parasite that efficiently infects DCs, which are then instrumental in presenting parasite antigens to prime *T. gondii*-specific T cell responses (Mashayekhi et al., 2011; Dupont et al., 2014). After infection, the parasite remains confined inside a parasitophorous vacuole (PV), which is actively remodeled. From this niche, the parasite establishes several interactions with the host cell in order to optimally replicate (Coppens and Romano, 2018). In this sense, *T. gondii* is capable of incorporating cholesterol and fatty acids from endolysosomes and lipid droplets (LDs). Indeed, *T. gondii* replication is inhibited by disrupting cholesterol transport (Coppens et al., 2000). Other studies show that the cholesterol level in the host cell impacts on *T. gondii* replication (Bottova et al., 2009; Hu et al., 2017; Nolan et al., 2017; Sanfelice et al., 2017). Moreover, the complexity of parasite-host interactions and their impact on the modulation of antigen presentation (Poncet et al., 2019), make *T. gondii* an interesting model of study. For example, the relevance of Sec22b in driving retrotranslocation of *T. gondii*-derived antigens from the PV lumen to the host cytosol, mediating the ER-associated degradation machinery recruitment, has been demonstrated (Cebrian et al., 2011). However, membrane-associated antigen cross-presentation has been shown to be independent of Sec22b, suggesting that the biochemical nature of the antigens and their disposition in the PV determines their processing pathway (Buaillon et al., 2017). Furthermore, the interaction with recycling endosomes, mediated by Rab22a, has also been shown to be necessary for efficient cross-presentation (Cebrian et al., 2016). Regarding the presentation of antigens in MHC-II, this pathway seems important to keep parasite proliferation under control and develop the chronic phase of infection (Leroux et al., 2015), but the mechanism by which *T. gondii*-derived antigens reach degradative compartments to be presented by MHC-II, it has not been completely defined (Poncet et al., 2019). The complexity of host-parasite interactions demands new approaches to understand how *T. gondii* modulates the functions of DCs.

In this work, we studied the impact of cholesterol trafficking on MHC-I and MHC-II antigen presentation. For this, we used the inhibitor U18666A, which interrupts the intracellular traffic of cholesterol by blocking the function of the NPC1 protein, leading to cholesterol accumulation in the endolysosomal compartments (Lu et al., 2015). We show that both MHC-II presentation and MHC-I cross-presentation were strongly disrupted in the presence of the inhibitor. Strikingly, the inhibitor not only blocks the presentation of *T. gondii*-associated antigens but also parasite proliferation. By EM analyzes, we observed intraluminal vesicles in the PVs. Since MVB biogenesis is inhibited by U18666A (Cenedella, 2009), we analyzed in *T. gondii* infected BMDCs the ESCRT machinery responsible for the generation of intraluminal vesicles. We observed that the main ESCRT-III component CHMP4b was massively recruited to the PV, and its expression level was upregulated upon *T. gondii* infection. U18666A treatment disrupted both the delivery of cholesterol and the recruitment of CHMP4b to the PV. Taken together, these results suggest an important role of cholesterol trafficking in antigen presentation, and on the proliferation of *T. gondii* within a functional PV.

## Materials and Methods

### Cells

C57BL/6 mice from 6 to 10 weeks of age were used to obtain bone marrow stem cells from the femur and tibia. Animals were maintained in specific pathogen-free conditions (SPF), housed in temperature-controlled rooms (22–25°C), and received water and food *ad libitum*. All animal procedures were performed according to the bioethics rules of the “Comité Institucional para el Cuidado y Uso de Animales de Laboratorio (CICUAL), Facultad de Ciencias Médicas, Universidad Nacional de Cuyo”. Cells were maintained in IMDM medium supplemented with 10% FBS and GM-CSF-containing supernatant to stimulate bone marrow-derived dendritic cell (BMDC) differentiation. After 9–14 days, immature BMDCs were used for experimental work. The GM-CSF-producing cell line J558 was kindly provided by Dr. Sebastian Amigorena (INSERM U932, Institute Curie, France). OT-IIZ, B3Z and BTg01Z (Grover et al., 2012) hybrid T cells were cultured with RPMI medium with 10% FBS. The different *T. gondii* strains were grown and maintained by infecting monolayers of HFF cells in DMEM complete medium. Intracellular parasites were recovered after HFF disruption by the use of a 23-G needle.

### Reagents

The following reagents were used in this study: Ovalbumin (OVA), lyophilized powder (Worthington Biochemical Corporation); Bovine Serum Albumin (BSA), lyophilized powder (Santa Cruz); 3 µm latex beads and 3 µm blue latex beads (Polysciences Inc.); OVA conjugated to Alexa Fluor 488, Fluoromount-G with DAPI (Invitrogen); Dako Omnis without DAPI (Agilent); IMDM, DMEM and RPMI media (Gibco); poly-L-lysine, saponin, sucrose, protease inhibitor cocktail, filipin, and DMSO (Sigma-Aldrich); Tricine, Tris Base, TEMED, and U18666A (Calbiochem); glycine (Bio-Rad); acrylamide (Promega); Ponceau S solution (Abcam); Imidazole and NP-40 (ICN Biomedicals Inc.); Fetal Bovine Serum (FBS) was purchased in Natocor-Industria Biológica (Argentina); CPRG (Roche Diagnostic GmbH); ToPro3 (Molecular Probes). Synthetic peptides: OVA_(257–264)_ SIINFEKL and OVA_(323–339)_ ISQAVHAAHAEINEAGR (Polypeptide Group); and CD4Ag28m_(605–619)_ AVEIHRPVPGTAPPS (Genecust).

### Antibodies

The following antibodies were used in this study: purified rabbit polyclonal anti-OVA (Sigma-Aldrich), purified FITC mouse anti-H-2K^b^ and PE mouse anti-IA^b^ (BD Pharmingen), rabbit polyclonal anti-Syntaxin 4 (Synaptic Systems), rabbit polyclonal anti-Rab11a (Aviva Systems Biology), rabbit polyclonal anti-CHMP4b and mouse monoclonal anti-TSG101 (Abcam), mouse monoclonal anti-SAG1 and rabbit polyclonal anti-CD63 (Santa Cruz). Purified rabbit anti-HPERVNVFDY (type I GRA6) and anti-GRA2 (Biotem). Anti-species conjugated to Alexa 488, 568, or 647 (Molecular Probes) or peroxidase (Jackson Laboratories) were used as secondary antibodies.

### Antigen Presentation Assays

**FIGURE 1:**
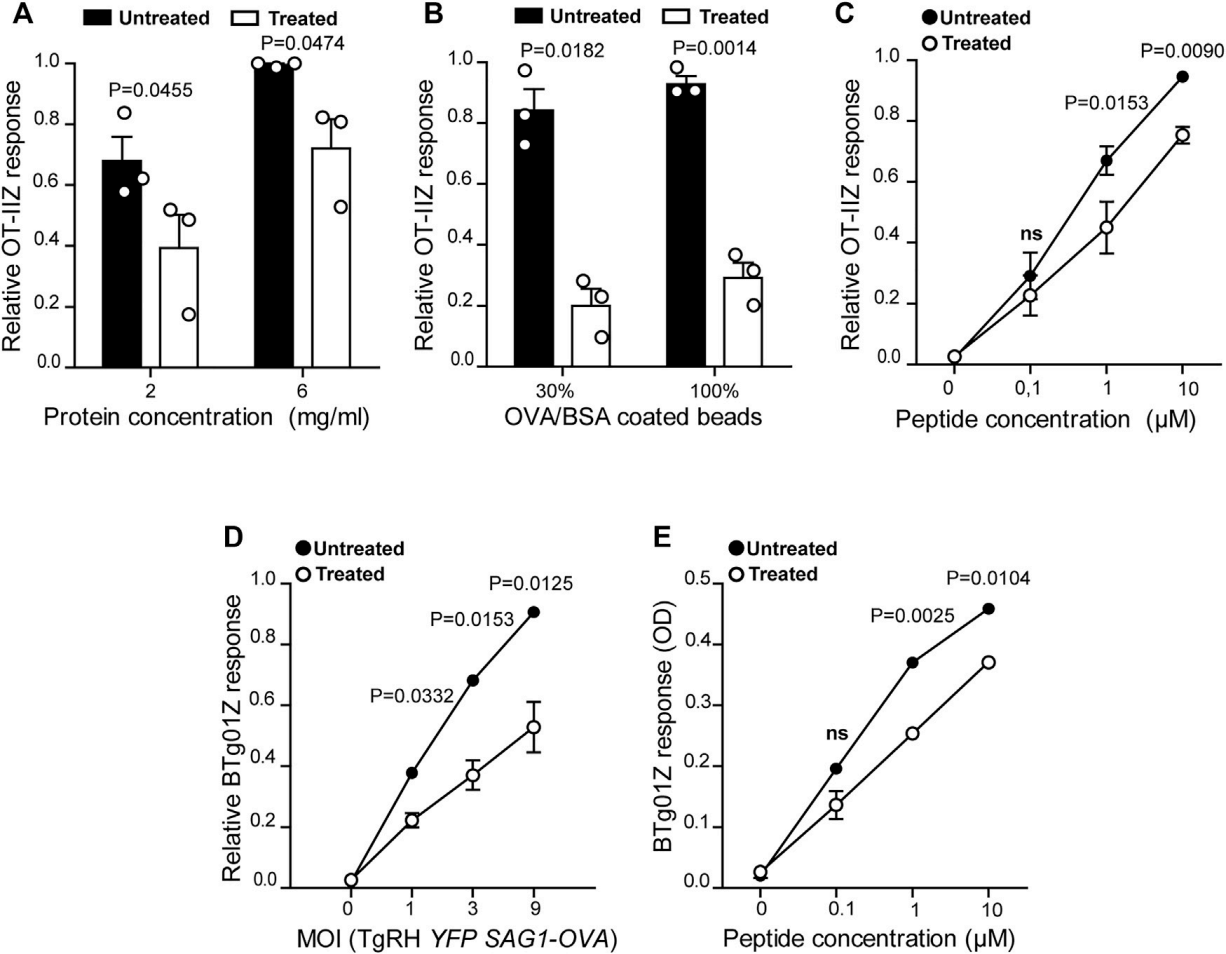
MHC-II antigen presentation is hampered in U18666A-treated BMDCs. MHC-II antigen presentation ability of treated and untreated BMDCs was evaluated by OT-IIZ cell activation after the incubation with **(A)** soluble OVA, **(B)** OVA/BSA coated latex beads, and **(C)** the ISQAVHAAHAEINEAGR control peptide at the indicated concentrations. **(D)** The presentation of a natural antigen derived from the parasite TgRH *YFP SAG1-OVA* after 8 h of infection at the indicated MOI and **(E)** the AVEIHRPVPGTAPPS control peptide at the indicated concentrations, was evaluated by BTg01Z cell activation. In **(A–** **D)**, data represent mean ± SEM of triplicate values from three independent experiments. In panel **(E)**, data show mean ± SEM of triplicate values of a single experiment. The *P-*value for each experimental condition is indicted in figure. *p* > 0.05 (ns). The one-tailed Student’s paired *t*-test was performed.

**FIGURE 2:**
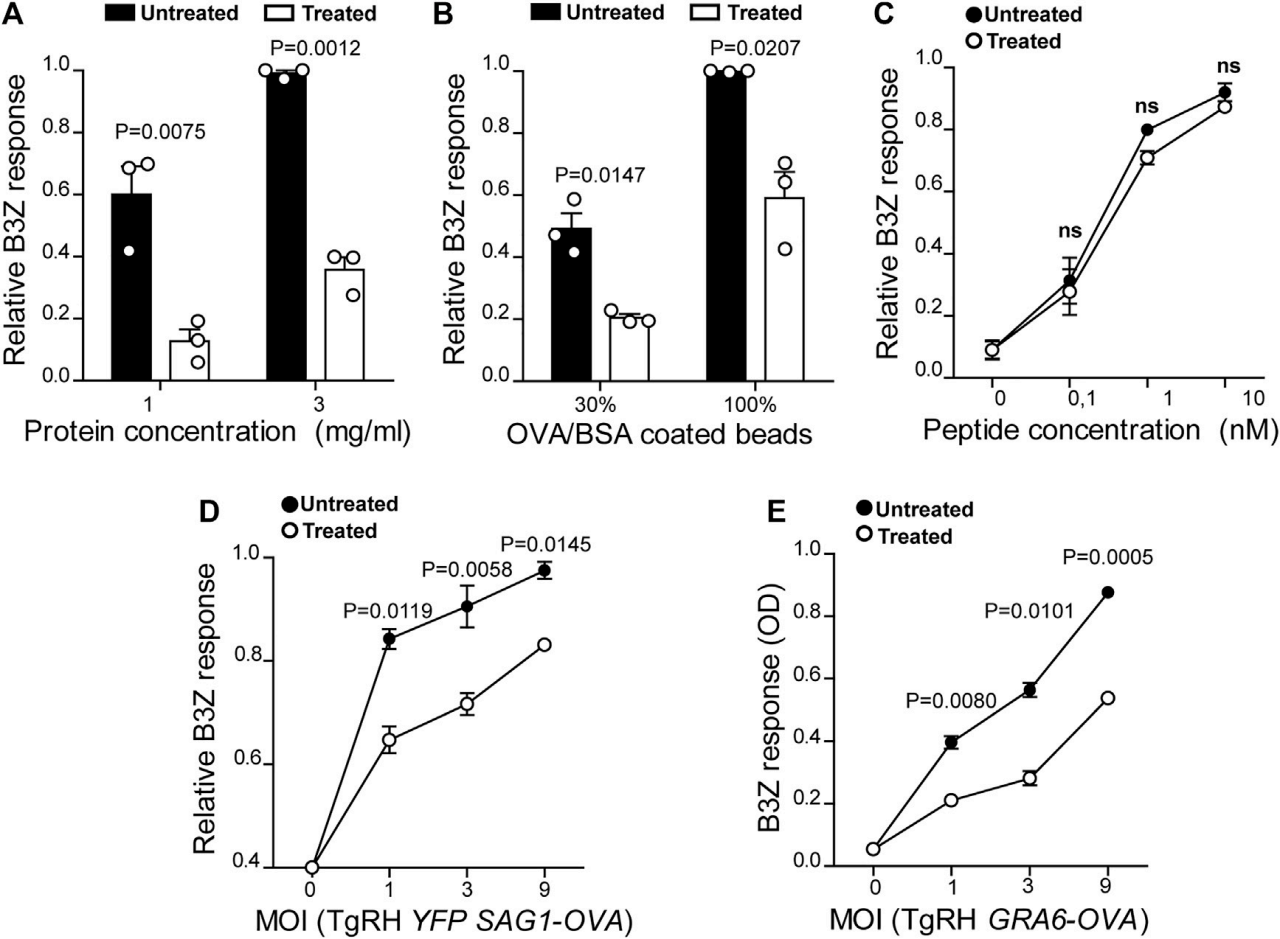
U18666A treatment inhibits antigen cross-presentation by BMDCs. The cross-presentation of **(A)** soluble OVA, **(B)** OVA/BSA coated latex beads, and **(C)** the SIINFEKL control peptide at the indicated concentrations by treated and untreated BMDCs was evaluated with the B3Z hybridoma. **(D)** MHC-I presentation of OVA secreted by TgRH *YFP SAG1-OVA* and **(E)** the SIINFEKL peptide appended at the C-terminus of the GRA6 antigen expressed by TgRH *GRA6-OVA*, after 8 h of treated/untreated BMDCs infection at the indicated MOI was evaluated by B3Z activation. In **(A–** **D)**, data represent mean ± SEM of triplicate values from three independent experiments. In panel E, data show mean ± SEM of triplicate values of a single experiment. The *P-*value for each experimental condition is indicted in figure. *p* > 0.05 (ns). The one-tailed Student’s paired *t*-test was performed.

**FIGURE 3:**
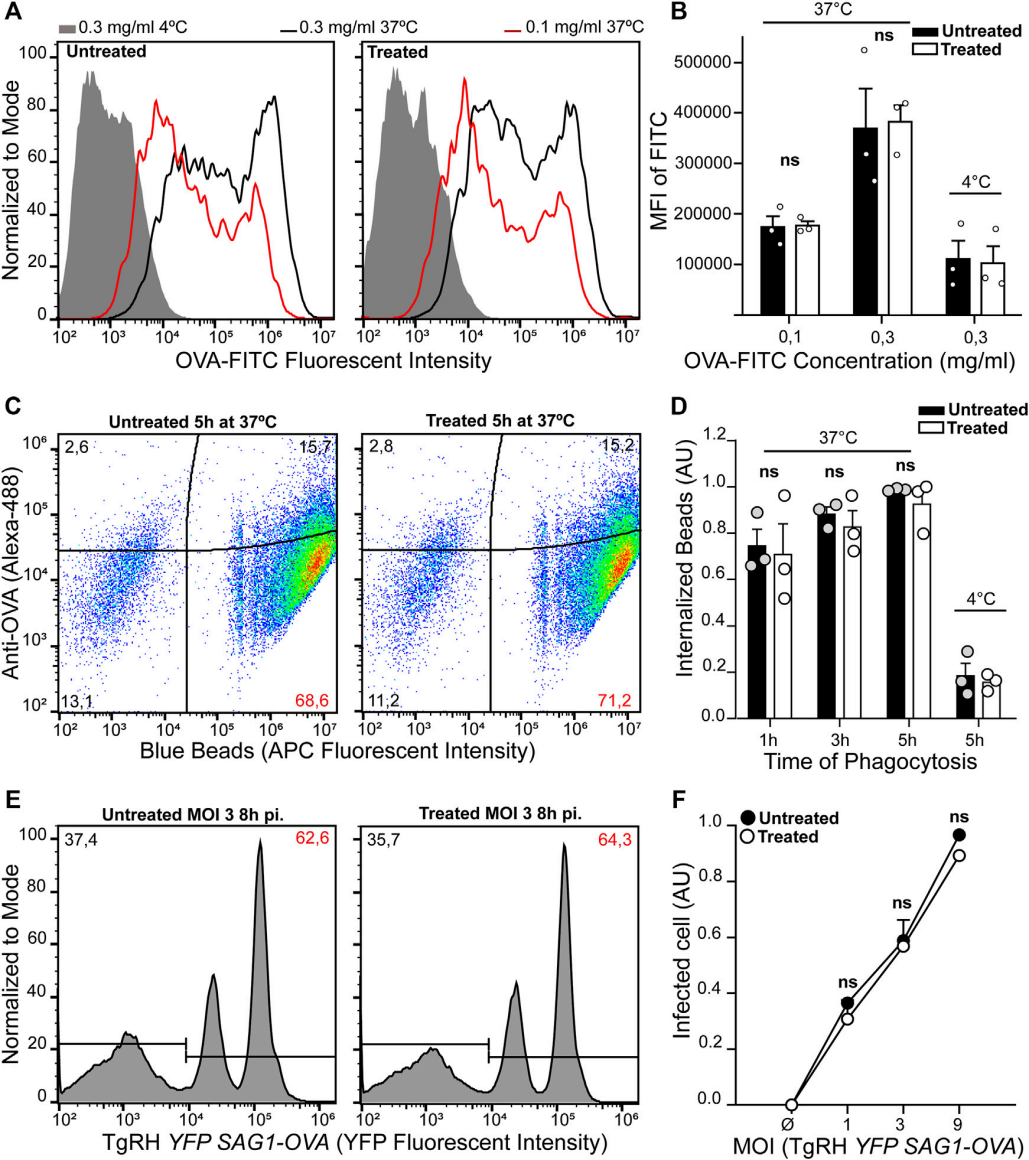
BMDC treatment with U18666A does not affect their antigen internalization capacity. **(A–** **D)** Evaluation of endocytosis and phagocytosis in untreated and treated BMDCs. Fluid-phase endocytosis of fluorescent OVA after 1 h of internalization, and phagocytosis of 3 µm fluorescent latex beads at different times of internalization were assessed by FACS analysis. Antigen internalization was performed at 37°C for effective uptake, and at 4°C as negative control. **(A)** Representative FACS profiles of OVA-FITC fluorescent intensity corresponding to the endocytosis of 0.3 mg/ml (black lines), 0.1 mg/ml (red lines) or endocytosis of 0.3 mg/ml at 4°C (gray lines). **(B)** Histogram showing the mean fluorescence intensity of FITC in untreated and treated BMDCs. Data represent mean ± SEM of triplicate values from three independent experiments. **(C)** Representative FACS profiles of untreated and treated BMDCs after 5 h of phagocytosis with 3 µm fluorescent latex beads coated with OVA. Red numbers indicate the percentage of cells that have completely internalized particles (APC+/OVA−). **(D)** Histogram showing the normalized percentage (arbitrary units, AU) of efficient phagocytosis after 1, 3, and 5 h of latex beads internalization at 37°C, and 5 h at 4°C. Data represent mean ± SEM of triplicate values from three independent experiments. **(E,F)** The efficiency of untreated and treated BMDCs infection for 8 h with TgRH *YFP SAG1-OVA* was measured by FACS analysis at the indicated MOI. **(E)** Representative FACS profiles of infected BMDC at MOI 3. **(F)** Histograms showing the normalized percentage of infected cells as arbitrary units (AU). Data represent mean ± SEM of triplicate values from three independent experiments. The one-tailed Student’s paired *t*-test was performed. *p* > 0.05 (ns).

For all antigens tested, BMDCs were treated with 7.5 μg/ml of U18666A (or same volume of DMSO) for a total period of 8 h at 37°C. In the case of experiments involving *T. gondii*, BMDCs were incubated with the U18666A inhibitor during the whole infection period (8 h). The parasite strains TgRH *YFP SAG1-OVA* or TgRH *GRA6-OVA* were used at the indicated MOI. For soluble OVA (specific concentrations indicated in Figures 1A, 2A) or 3 µm latex beads coated with different ratios of OVA and BSA (10 mg/ml of OVA alone, 3 mg/ml of OVA and 7 mg/ml of BSA or 10 mg/ml of BSA alone), BMDCs were first pre-treated with U18666A for 3 h and then incubated 5 h more with the mentioned antigens. For the short control peptides ISQAVHAAHAEINEAGR (OVA 323–339 for OT-IIZ cells), AVEIHRPVPGTAPPS (AS15 for BTg01Z cells) or SIINFEKL (OVA 257-264 for B3Z cells), BMDCs were pre-treated for 6 h with U18666A plus 2 h with the corresponding peptide. After incubation, cells were washed three times with 0.5% BSA/PBS, fixed with 0.008% glutaraldehyde during 2 min at 4°C and quenched with 0.2M glycine. Finally, one final wash with PBS was performed, and the corresponding T cell hybridoma was added during 16 h at 37°C. T cell activation was colorimetrically determined detecting ß-galactosidase activity by optical density (absorbance at 595–655 nm) using CPRG as substrate for the reaction. When indicated, relative T cell response corresponds to the ratio between optical density of each experimental condition and the mean of optical densities of the highest antigen concentration of untreated cells, and it was expressed as arbitrary units (AU).

### FACS Experiments

#### Cell Viability

To test cell viability after treatment, cells were incubated during 24 h in complete medium with 7.5 μg/ml of U18666A (or the same volume of DMSO) at 37°C. Then, cells were washed twice in PBS and stained with ToPro3 (1:3000) and immediately analyzed by flow cytometry. The population was selected and the doublets eliminated, using the gating strategy show in Supplementary Figure S2A, to determine the percentage of viable BMDCs (ToPro3 negative cells).

#### MHC-I and MHC-II Staining

BMDCs were incubated for 24 h in complete medium with 7.5 μg/ml of U18666A, or the same volume of DMSO. Then, cells were fixed with 2% PFA for 10 min at 4°C and quenched with 0.2M glycine. After this, cells were permeabilized with permeabilization buffer (0.05% saponin/0.2% BSA/PBS) for 20 min at RT, washed and incubated with FITC mouse anti-H-2K^b^ (MHC-I) or PE mouse anti-IA^b^ (MHC-II) for 40 min at 4°C. To measure the cell surface expression of MHC molecules, intact cells were used (without fixing or permeabilizing). Finished staining, cells were washed three times with permeabilization buffer (or with PBS/0.5% BSA for intact cells), twice with PBS and mean fluorescent intensity (MFI) was obtained by flow cytometry analysis. MFI values were normalized to the mean of each control and expressed as AU.

#### Antigen Uptake and *T. gondii* Infection Assay

To determine the endocytic capacity of BMDCs in presence of the inhibitor, cells were pre-treated with 7.5 μg/ml of U18666A (or the same DMSO volume) during 7 h and incubated for 1 h more at 37°C with 0.1 or 0.3 mg/ml of OVA coupled to FITC in complete medium. To control unspecific binding of OVA-FITC, cells were incubated with the highest concentration of this fluorescent antigen at 4°C. Then, BMDCs were washed three times with 0.5% BSA/PBS and the MFI of FITC was determined by flow cytometry analysis.

To evaluate the phagocytic capacity of BMDCs, 3 μm blue latex beads were previously coated with 10 mg/ml of OVA. Cells were treated with 7.5 μg/ml of U18666A, or the same DMSO volume, during 8 h and incubated for 1, 3 or 5 h at 37°C with the OVA-coated particles in complete medium. Control and treated BMDCs were also incubated for 5 h at 4°C with OVA-coated beads as negative control of phagocytosis. After each internalization period, cells were washed three times with 0.5% BSA/PBS, incubated 40 min at 4°C with a rabbit polyclonal anti-OVA antibody, then washed and labeled with a secondary anti-rabbit antibody coupled to Alexa 488 also 40 min at 4°C. After three final washes with 0.5% BSA/PBS, cells were analyzed by flow cytometry, as shown in Figure 3C. The percentage of internalized beads was normalized to the mean of 5 h phagocytosis of untreated cells and expressed as AU.

To test *T. gondii* infection upon U18666A treatment, BMDCs were incubated during 8 h at 37°C with the fluorescent strain TgRH *YFP SAG1-OVA* at MOI 1, 3 or 9 in complete medium with 7.5 μg/ml of inhibitor, or the equivalent DMSO volume. Then, cells were extensively washed with 0.5% BSA/PBS, fixed with 2% PFA during 10 min at 4°C, washed twice with 0.2M glycine, and one final wash with 0.5% BSA/PBS. The infection rate of BMDCs was determined by FACS analysis, as shown in Figure 3E. The percentage of infected cells was normalized to the mean of the condition “MOI 9” of untreated cells and expressed as AU.

### Confocal Microscopy

BMDCs were cultured on poly-L-lysine-coated glass coverslips during 24 h in complete medium with 7.5 μg/ml of U18666A (or the same volume of DMSO). After extensive washing with PBS, BMDCs were first fixed with 2% PFA during 15 min at 37°C and then quenched with 0.2M glycine. After this, cells were permeabilized with 0.05% saponin/0.2% BSA/PBS for 20 min at RT, washed and incubated with mouse anti-H-2K^b^ (MHC-I) or mouse anti-I-A^b^ (MHC-II), combined with rabbit anti-Syn4, overnight at 4°C. The combinations of mouse anti-H-2K^b^ (MHC-I) plus rabbit anti-Rab11a, and mouse anti-I-A^b^ (MHC-II) plus filipin were also used. The next day, cells were washed with permeabilization buffer and incubated with a secondary antibody coupled to Alexa 488 or Alexa 555 for 60 min at 4°C. Cells were washed again three times with permeabilization buffer and twice with PBS. Finally, coverslips were mounted with Fluoromount-G (with DAPI).

To observe *T. gondii* infection, BMDCs were incubated at 37°C with TgRH *YFP SAG1-OVA* at MOI 1 on poly-L-lysine-coated glass coverslip for the indicated time in each section. Subsequently, cells were fixed and permeabilized the same way as for non-infected BMDCs. To detect endogenous MVBs component recruited to the PV, cells were incubated with rabbit anti-CD63, mouse anti-TSG101 or rabbit anti-CHMP4b, combined with mouse anti-SAG1, rabbit anti-GRA6 or mouse anti-GRA2, respectively, overnight at 4°C. Next, cells were washed with permeabilization buffer and incubated with secondary antibodies coupled to Alexa 555 or Alexa 647 for 60 min at 4°C. Finally, cells were washed and mounted as described before for non-infected cells.

For cholesterol labeling, *T. gondii* infected (TgRH *YFP SAG1-OVA* at MOI 1) or non-infected BMDCs were incubated with 1 mg/ml of filipin for 1 h at RT, prior to fixing, and mounted with Dako Omnis mounting medium without DAPI.

Image acquisition was performed on an Olympus FV-1000 confocal microscope with a 63×/1.4 NA oil immersion objective. One z-stack plane is shown from the acquired images and they were processed with the ImageJ software (Wayne Rasband, National Institutes of Health). Image deconvolution was performed with the Parallel Spectral Deconvolution plugin (Piotr Wendykier) using a theoretical PSF generated by the Diffraction PSF 3D plugin (Robert Dougherty).

### Immunoblotting

BMDCs were incubated with TgRH *YFP SAG1-OVA* at MOI 2 and 6 in complete medium, during 2, 8 or 24 h at 37°C. Then, 10^5^ cells were washed three times with PBS and resuspended in 20 μl of sample buffer (50 mM Tris, 4% SDS, 9.5% glycerol and 2% β-mercaptoethanol). Total cell lysates were subjected to SDS-PAGE on 12% gel. After transferring, the membrane was stained with Ponceau S and washed with distilled water until all the dye was removed. Next, the membrane was blocked in 10% Milk/PBS during 1 h at RT and incubated with anti-CHMP4b, anti-SAG1 and then with peroxidase-conjugated antibodies. Bound antibodies were revealed using the kit Chemiluminescent Peroxidase Substrate-3 (Sigma-Aldrich), according to the manufacturers’ instructions. The intensity of the bands was quantified by densitometry using Quantity One 4.6.6 software (Bio-Rad) and was expressed as arbitrary units.

### Transmission Electron Microscopy

BMDCs were infected with TgRH *YFP SAG1-OVA* at MOI 1 during 24 h at 37°C. Then, cells were washed with ultrapure PBS three times, and fixed with 2.5% glutaraldehyde during 1 h at 4°C. Cells were washed again three times with ultrapure PBS (5 min at 4°C each), and incubated in 1% osmium tetroxide/PBS for 2 h at RT. Then they were dehydrated sequentially with increasing concentrations of ice-cold acetone and three times with 100% acetone for 15 min at RT. Cells were infiltrated in 1:1 acetone:EPON overnight at RT and finally embedded in fresh pure resin overnight at RT. Thin sections (60–80 nm) were cut with a diamond knife (Diatome, Washington, DC) on a Leica Ultracut R ultramicrotome and collected on 200-mesh copper grids. Grids were observed and photographed in a Zeiss 902 electron microscope at 50 kV.

### Focused Ion Beam and Scanning Electron Microscopy (FIB-SEM)

HFF were brought to confluence on a plate with grid coverslip, infected for 24 h with TgRH *YFP SAG1-OVA* and fixed overnight at 4°C in 2.5% glutaraldehyde in 100 mM Hepes pH 7.4. Cells were incubated with 1% osmic acid, 1.5% potassium ferrocyanide in Hepes for 1 h at RT, then with 1% tannic acid in Hepes for 30 min at RT followed by 1% osmic acid in H_2_O for 1 h at RT. Samples were dehydrated using ethanol gradient of 25, 50, 75, 95% for 10 min each, then 100% three times for 15 min. Cells were infiltrated in EPON overnight at RT and embedded in fresh pure resin for 2 h at RT, left to polymerize for 48 h at 60°C. Consecutive face-block imaging and milling was performed on a Zeiss Crossbeam 540. Voxel size of the xy-binned images is 10 × 10 × 5 nm (xyz).

### Statistical Analysis

The one-tailed Student’s paired *t*-test was performed at the indicated Figures by using the GraphPad Prism 5 software. The ImageJ software was used for imaging processing.

## Results

### U18666A Treatment Impairs Exogenous Antigen Presentation by BMDCs

To confirm the critical importance of cholesterol transport for MHC-II antigen presentation by DCs (Anderson et al., 2000; Bosch et al., 2013b; Roy et al., 2013), we tested the impact of the inhibitor U18666A on this immune process. Therefore, we evaluated MHC-II antigen presentation by the use of OT-IIZ CD4+ T cells, which specifically recognizes the 17 amino acids sequence (ISQAVHAAHAEINEAGR) derived from ovalbumin (OVA), commonly known as 323–339 peptide, loaded onto I-A^b^ MHC-II molecules. We treated BMDCs with 7.5 μg/ml of U18666A, or the equivalent DMSO volume for the control condition, and incubated these cells with soluble OVA or OVA coupled to 3 μm latex beads for 5 h at 37°C. As shown in Figures 1A,B, U18666A treatment induced a strong reduction of CD4+ T cell activation in the context of endocytosis and phagocytosis, respectively. Also, the presentation of the short control 323–339 peptide, which does not require further processing to associate with MHC-II molecules, was significantly decreased in U18666A-treated BMDCs (Figure 1C). Consistent with previous reports, this result may reflect a reduced stability of MHC-II/peptide complexes at the cell surface due to cholesterol depletion (Bosch et al., 2013b). We next addressed the impact of U18666A treatment on the presentation of *T. gondii*-derived antigens. For this, we used a different CD4+ T hybridoma called BTg01Z, which recognizes a natural antigen of the parasite. BTg01Z cells are activated in response of recognizing the AS15 peptide (AVEIHRPVPGTAPPS) of the CD4Ag28m *T. gondii* protein loaded onto I-A^b^ MHC-II molecules at the plasma membrane of antigen-presenting cells (Grover et al., 2012). As shown in Figure 1D, BTg01Z CD4+ T cell activation was strikingly affected in the presence of U18666A after 8 h of *T. gondii* infection. Also in this experimental setup, the presentation of the corresponding short peptide was significantly inhibited after U18666A treatment (Figure 1E). These results indicate that correct cholesterol transport is necessary for adequate MHC-II antigen presentation.

DCs are highly adapted to process and present exogenous antigens in MHC-I molecules through the cross-presentation pathway. Although these cells require cholesterol in the plasma membrane to facilitate antigen internalization by macropinocytosis (Albrecht et al., 2006), the role of cholesterol trafficking in the context of cross-presentation remains unknown. To address this question, we treated BMDCs with U18666A and we used B3Z CD8+ T cells, which specifically recognizes the OVA-derived SIINFEKL peptide in association with H-2K^b^ MHC-I molecules. As shown in Figures 2A,B, U18666A-treated BMDCs fail to cross-present both soluble and particulate antigens, respectively. However, in this case no significant differences of CD8+ T activation were observed between control and treated cells after incubation with the short SIINFEKL peptide (Figure 2C). Similar to MHC-II presentation, the cross-presentation of *T. gondii*-derived antigens was impaired in U18666A-treated BMDCs after infection with TgRH *YFP SAG1-OVA* parasites, which secrete the model antigen OVA as a soluble protein into the vacuolar space (Figure 2D). Given that the distribution of *T. gondii* antigens inside the PV also determines the pathway followed by MHC-I molecules to activate CD8+ T lymphocytes (Buaillon et al., 2017; Poncet et al., 2019), we decided to analyze the presentation of a transmembrane antigen. Thus, we used the strain TgRH *GRA6-OVA*, which expresses a portion of OVA fused with the parasite membrane and immunodominant antigen GRA6 (Blanchard et al., 2008). Here again, MHC-I presentation of the GRA6-OVA antigen was significantly impaired in U18666A-treated BMDCs, as compared to control cells (Figure 2E). Altogether, these data show that cholesterol transport represents a key aspect of DC intracellular trafficking in order to carry out optimal MHC-II presentation and MHC-I cross-presentation of exogenous antigens.

### U18666A Treatment Does Not Affect Cell Viability or Antigen Internalization by BMDCs

The drug U18666A inhibits cholesterol intracellular transport leading to an accumulation of this lipid within lysosomes (Cenedella, 2009; Lu et al., 2015). Since cholesterol is a key component of cell membranes, vital functions could be altered in treated DCs, thereby generating the defective phenotype of exogenous antigen presentation that we observe in our system. Therefore, we controlled the viability of U18666A-treated and untreated BMDCs. We used the fluorescent nuclear probe ToPro3 that binds to dead cell nucleus, but cannot access the DNA of viable cells. Cells were treated with the U18666A inhibitor (or the equivalent volume of DMSO) at 37°C for 24 h, a longer incubation period compared to the one used in the antigen presentation assays. In Supplementary Figures S1A,B is depicted the flow cytometry gating strategy for this experimental approach. Our results show that no significant differences of cell viability were found between control and U18666A-treated BMDCs (Supplementary Figure S1C).

Next, we decided to analyze the uptake capacity of exogenous antigens after U18666A treatment, since a defect at this level would determine the efficiency of CD4+/CD8+ T cell activation. After 7 h of U18666A treatment, BMDCs were incubated with different concentrations of OVA coupled to FITC for 1 h at 37°C, or the highest concentration of this antigen at 4°C, and the mean fluorescent intensity (MFI) of FITC was analyzed by flow cytometry. As shown in Figures 3A,B, no significant differences in fluid-phase endocytosis were found between control and U18666A-treated BMDCs.

Next, the phagocytic capacity of U18666A-treated BMDCs was evaluated by incubating these cells with 3 µm OVA-coated fluorescent latex beads for 1, 3, and 5 h. Again, the total period of U18666A treatment was 8 h. After each uptake time point, BMDCs were labeled with an anti-OVA antibody in order to discriminate between fluorescent particles attached to the cell surface (APC+/Alexa 488+) from those that were fully internalized (APC+/Alexa 488−), as shown in Figure 3C. As a control, we also incubated BMDCs with OVA-coated fluorescent beads for 5 h at 4°C to inhibit phagocytosis. We quantified the percentage of cells that fully internalized the particulate antigen in all experimental conditions, and did not find significant differences between control and U18666A-treated BMDCs (Figure 3D).

Finally, we investigated the infection rate by *T. gondii* in the presence or absence of the inhibitor. BMDCs were treated with U18666A or DMSO for 8 h and co-incubated with the fluorescent parasite strain TgRH *YFP SAG1-OVA* at different multiplicity of infection (MOI). Figure 3E shows representative flow cytometry profiles exhibiting uninfected cells (populations on the left), and BMDCs with one or two fluorescent parasites inside (populations on the right). As shown in Figure 3F, we quantified the percentage of infected cells by *T. gondii*, and no significant differences were observed between control and U18666A-treated BMDCs.

This set of experiments indicates that U18666A treatment does not affect fluid-phase endocytosis, phagocytosis, *T gondii* infection or cell viability, suggesting that another important intracellular trafficking step is perturbed upon BMDC incubation with this inhibitor and that it cripples exogenous antigen presentation.

### U18666A Treatment Disrupts MHC-I and MHC-II Molecules Transport to the Plasma Membrane

**FIGURE 4:**
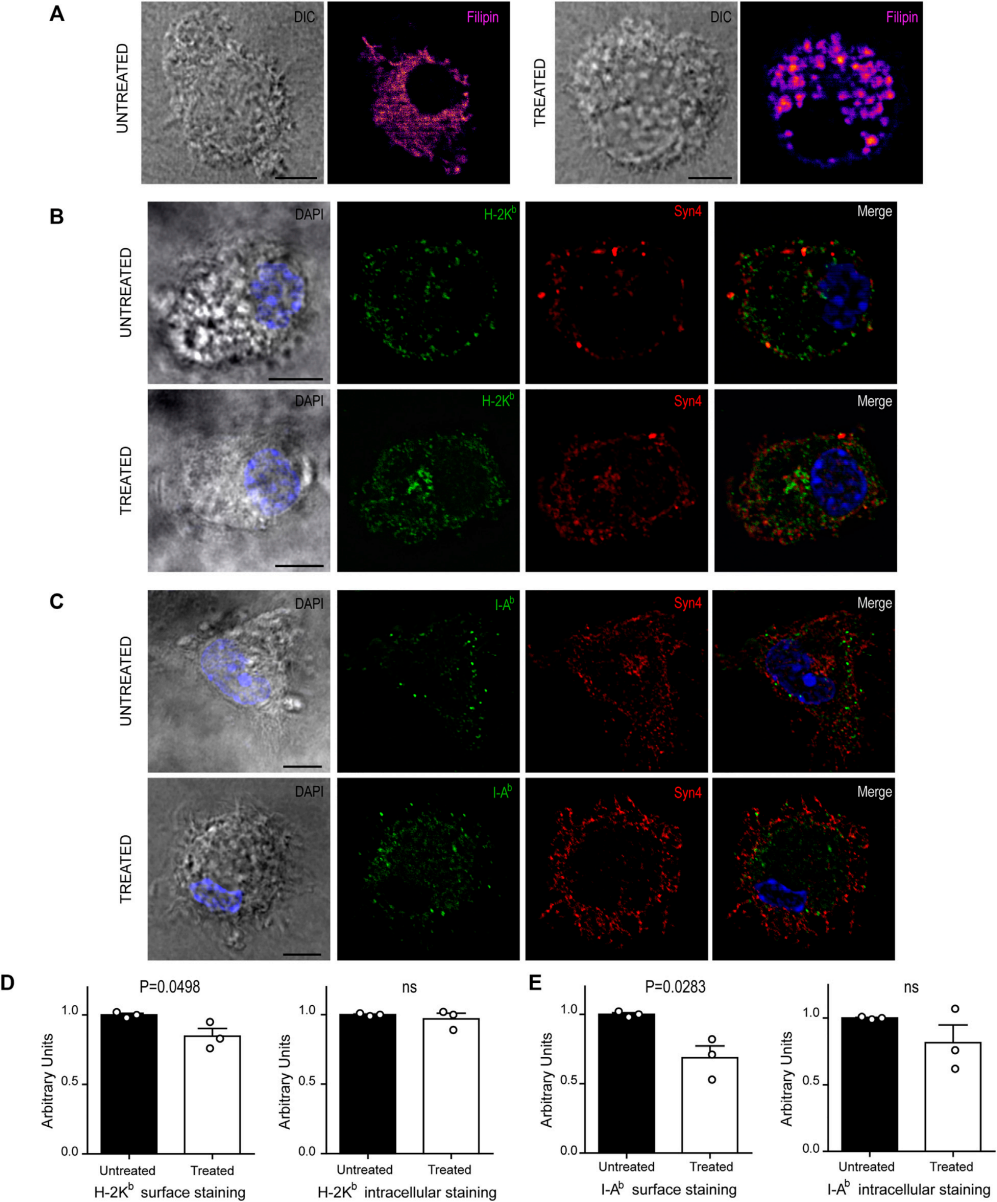
U18666A treatment disrupt the arrival of MHC-I and MHC-II molecules to the cell surface. **(A–C)** Immunofluorescence labeling and confocal microscopy analysis showing the distribution of cholesterol, MHC molecules and a surface marker, in untreated and treated BMDCs for 24 h. **(A)** Cholesterol stained with filipin (fire). **(B)** H-2K^b^ MHC-I (green) and Syn4 (red). **(C)** I-A^b^ MHC-II (green) and Syn4 (red). Nuclei stained with DAPI. DIC images are shown on the left. Overlay of all fluorescent channels is shown in the right panels. Scale bars: 5 µm. Data are representative of 10 images analyzed for each experimental condition from three independent experiments. **(D,E)** FACS analysis of MHC-I and MHC-II molecules of untreated or treated BMDCs. Intact cells corresponding to surface expression (left) and permeabilized cells corresponding to total expression of MHC molecules (right). Histograms showing arbitrary units (AU) corresponding to values normalized to the mean of each control. Data represent mean ± SEM of triplicates values from three independent experiments. The *P-*value for each experimental condition is indicted in figure. *p* > 0.05 (ns). The one-tailed Student’s paired *t*-test was performed.

We next decided to explore if U18666A treatment may be interfering with normal intracellular transport and distribution of MHC-I and MHC-II molecules in BMDCs, a key feature of antigen presentation. First, we analyzed cholesterol distribution in control and treated BMDCs by using filipin labeling and confocal microscopy. As shown in Figure 4A, control cells exhibited a uniform membrane distribution of cholesterol throughout the cytoplasm, whilst in U18666A-treated BMDCs, cholesterol was accumulated in a more marked patchy vesicular pattern, as described before in other cell types (Shoemaker et al., 2013; Elgner et al., 2016).

Since U18666A treatment interrupts cholesterol trafficking from lysosomes to the plasma membrane, we thought that the regular transport of MHC molecules may be also altered. Therefore, we labeled BMDCs after 24 h of U18666A treatment with specific fluorescent tagged antibodies that detect endogenous H-2K^b^ MHC-I and I-A^b^ MHC-II molecules. We also included the detection of endogenous Syntaxin 4 (Syn4), a plasma membrane SNARE protein that can be present in DC endosomes and phagosomes (Cebrian et al., 2011). As shown in Figures 4B,C, MHC-I and MHC-II molecules seem to be more accumulated at the intracellular level and less in the periphery of U18666A-treated BMDCs, as compared to control BMDCs, suggesting a defect of transport to the plasma membrane. A perinuclear structure positive for Syn4 was more evident upon U18666A treatment than in control cells, suggesting an intracellular accumulation of MHC-I molecules in this region. Therefore, we performed a double staining to detect H-2K^b^ and Rab11a, a classical marker of recycling endosomes that highly co-localizes with the intracellular pool of MHC-I molecules (Nair-Gupta et al., 2014). As shown in Supplementary Figure S2A, U18666A-treated BMDCs exhibit higher accumulation of MHC-I molecules in Rab11a-positive endosomes and a weaker staining at the cell surface, as compared to untreated cells. Moreover, we decided to label I-A^b^ and filipin given that MHC-II molecules and cholesterol are present within acidic compartments of the endocytic network. Indeed, Supplementary Figure S2B shows that U18666A treatment induces stronger co-localization of MHC-II molecules with filipin than in control BMDCs, indicating higher intracellular accumulation of these molecules in endolysosomes. Conversely, the cell surface staining of I-A^b^ MHC-II molecules is weaker in treated than in untreated BMDCs.

To confirm this observation, we quantified intracellular and cell surface-associated MHC-I and MHC-II molecules in treated and untreated BMDCs by flow cytometry analysis. For the cell surface staining, we performed experiments with intact BMDCs, while the intracellular stainings were done with cells previously fixed and permeabilized with saponin. This experimental setup confirms that indeed, the cell surface expression levels of both MHC-I and MHC-II molecules, were significantly decreased in U18666A-treated BMDCs, as compared to control BMDCs (Figures 4D,E, respectively). By contrast, no major changes of intracellular staining were observed between control and treated cells. Although a tendency of lower amounts of intracellular MHC-II molecules was observed in U18666A-treated cells, this difference was not statistically significant (Figure 4E). Representative FACS histograms are shown in Supplementary Figure S3.

These results suggest that BMDC treatment with U18666A impacts on exogenous antigen presentation, at least partially, by arresting cholesterol intracellular trafficking and optimal transport of MHC-I and MHC-II molecules to the cell surface.

### Intraluminal Vesicle Formation in the Vacuolar Space of *T. gondii* PV and CHMP4b Recruitment to the PV

**FIGURE 5:**
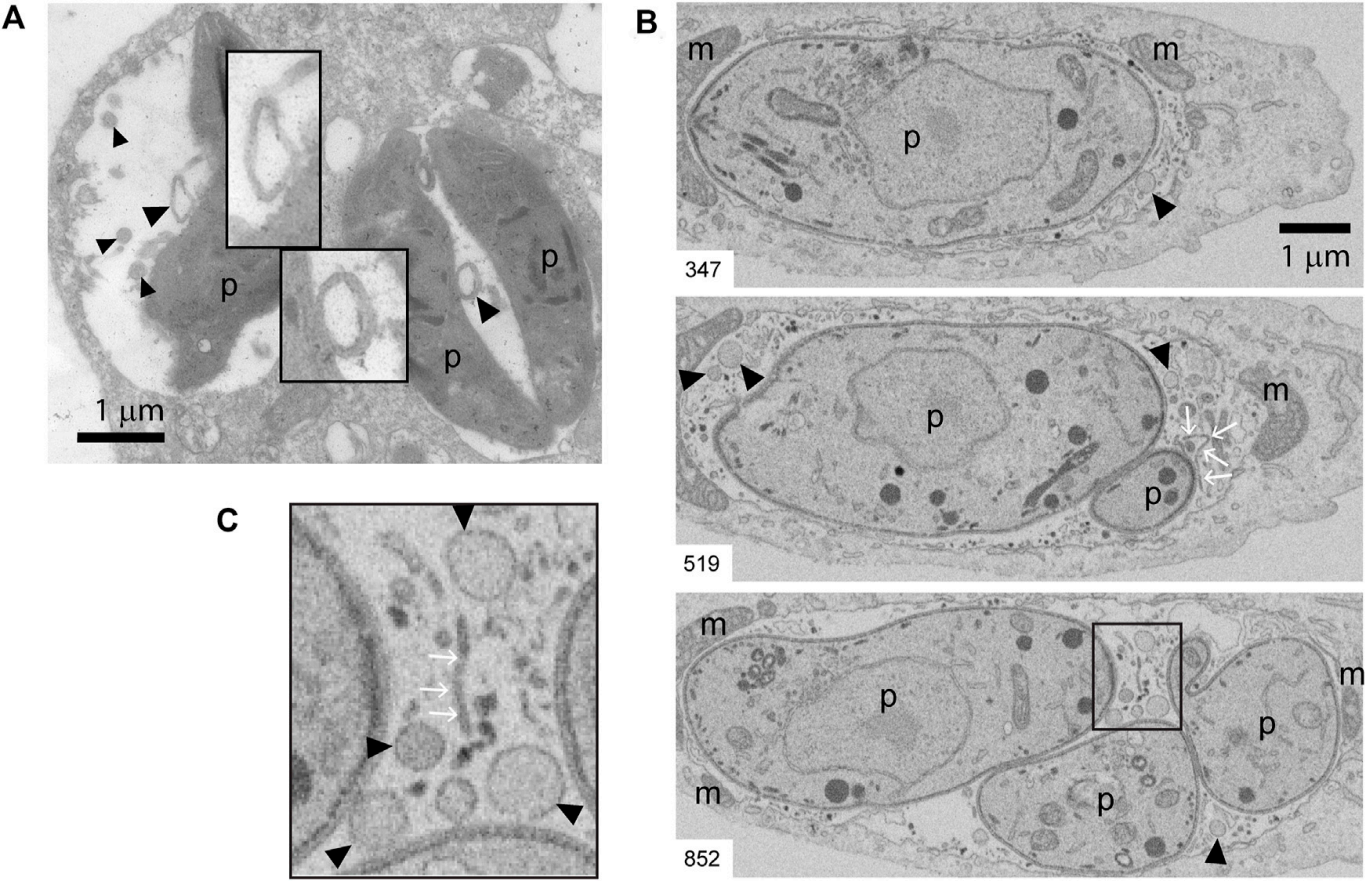
TEM and FIB-SEM showing intravacuolar vesicles in infected BMDCs. **(A)** Transmission EM of *T. gondii-*infected BMDCs at MOI 1 for 24 h. Black arrowheads indicate intraluminal vesicles inside PVs. Magnification denote double-membrane surrounded vesicles. Data are representative of 30 images acquired and analyzed from three independent experiments. **(B,C)** Representative FIB-SEM images of a 24 h-infected human fibroblast, extracted from the stack that is shown as Supplementary Figure S4 Movie. The number at the bottom left indicates the position of the section in the stack. Black arrowheads point to intraluminal vesicles, white arrows point to tubules. **(C)** Inset of magnified area taken from slice #852. m: host mitochondria closely apposed to PV limiting membrane, p: parasite. A total of four movies (stacks) from different infected cells were analyzed. Intraluminal vesicles were observed in all the stacks.

Beyond its clear influence on MHC molecule trafficking and antigen presentation, intracellular transport of cholesterol is likely to also impact the host-parasite membrane interface, and in turn regulate *T. gondii* fitness within the host cell. The host-parasite membrane interface comprises not only the PV limiting membrane but also an intravacuolar network (IVN) of membrane tubules that is involved in sequestration of *T. gondii* membrane-bound antigens (Lopez et al., 2015), and in ingestion of host-derived proteins (Dou et al., 2014) and vesicles (Romano et al., 2017). Beside the tubules, by analyzing PVs at the ultrastructural level, we noticed the presence of numerous round-shaped vesicles in the lumen of *T. gondii* vacuoles. Figure 5A shows transmission electron microscopy (TEM) images depicting these intravacuolar vesicles. Although they exhibit some similarity with the intraluminal vesicles present in multivesicular bodies (MVBs), they are larger and more heterogeneous in size. Indeed, we frequently found big-size vesicles that are shown with higher magnification in the insets. To enhance the visualization of such intraluminal vesicles over the entire vacuolar space, we took advantage of an EM tomography approach called Focused Ion Beam-Scanning Electron Microscopy (FIB-SEM). Acting as a ‘slicing nano-scalpel’, the ion beam mills a bulk sample, progressively exposing the deeper regions of the material. The exposed block face is then imaged at high resolution with the electron beam, resulting in a stack of hundreds of serial images that encompass the entire PV (Supplementary Figure S4 Movie). As illustrated on planes extracted from the stack (Figures 5B,C), this approach confirmed the presence of tubules and vesicles in the lumen of the PV.

**FIGURE 6:**
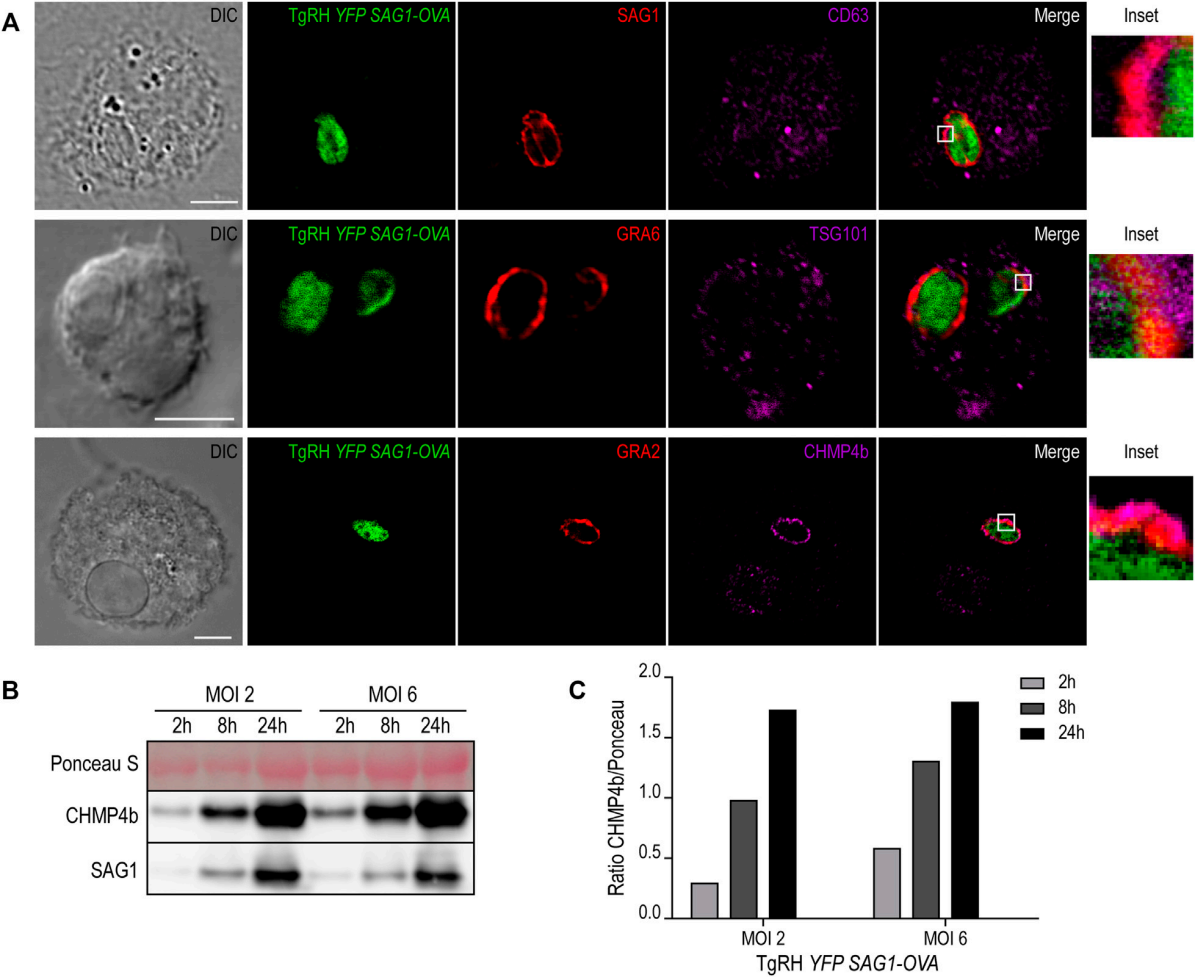
MVBs components recruitment to the PV and CHMP4b over-expression in infected BMDCs. **(A)** Immunofluorescence labeling and confocal microscopy analysis showing the parasite proteins (SAG1, GRA6 or GRA2) in red and MVBs markers (CD63, TSG101 or CHMP4b) in magenta, in BMDCs infected with TgRH *YFP SAG1-OVA* (green) for 8 h. DIC images are shown on the left. Overlay of all fluorescent channels is shown in the right panels. Scale bars: 5 µm. Data are representative of 30 images analyzed for each experimental condition from three independent experiments. **(B)** Immunoblotting showing CHMP4b and SAG1 expression of BMDCs infected by TgRH *YFP SAG1-OVA* at MOI 2 and MOI 6, for 2, 8, and 24 h pi. **(C)** Densitometry quantification of Ponceau S and CHMP4b of BMDCs infected by TgRH *YFP SAG1-OVA*, at MOI 2 and MOI 6, at 2, 8, and 24 h pi. Data are representative of three independent experiments.

The molecular machinery responsible of generating intraluminal vesicles in MVBs is composed of the endosomal sorting complexes required for transport (ESCRT) proteins. This family of proteins, in association with other accessory molecules, form cytosolic complexes that sequentially interact to induce membrane deformation, invagination and inward endosomal budding (Henne et al., 2011). We hypothesized that the host cell ESCRT machinery required for MVB biogenesis could be involved also in the formation of such *T. gondii* intravacuolar vesicles. To test this idea, we first studied the recruitment of different MVBs markers to the PV, such as the tetraspanin CD63, the ESCRT-I member TSG101, and the ESCRT-III protein CHMP4b. We infected BMDCs with the fluorescent *T. gondii* strain TgRH *YFP-SAG1/OVA* for 8h, and we labeled the parasite proteins SAG1, GRA6 and GRA2 in combination with endogenous CD63, TSG101 and CHMP4b, respectively. As shown in Figure 6A, all three markers visibly localized to the PV membrane, indicating that *T. gondii* efficiently intercepts MVB components during DC infection. For CD63 and TSG101 labeling, the vesicular pattern displayed in BMDCs at steady state (Supplementary Figures S5A,B, respectively) was maintained after *T. gondii* infection (Figure 6A, top and middle panels). However, CHMP4b recruitment to the PV was striking and this protein re-localized almost completely to the parasite vacuole upon *T. gondii* infection (Figure 6A, lower panel). Moreover, the vesicular distribution of CHMP4b throughout the cytoplasm was lost upon *T. gondii* infection (Supplementary Figures S5C,D). A total redistribution of CHMP4b was accompanied by an increase of the fluorescence intensity, suggesting that *T. gondii* infection induces an upregulation of CHMP4b expression.

To directly test if *T. gondii* infection promotes the accumulation of CHMP4b, BMDCs incubated with two MOI were homogenized after different times of infection. The extracts were analyzed by SDS-PAGE and probed with anti-CHMP4b and anti-SAG1 antibodies. Figures 6B,C confirms that CHMP4b is strongly induced upon infection, correlating with the accumulation of the *T. gondii* SAG1 protein over time. Therefore, there is a clear positive correlation between the amount of parasites inside BMDCs and the expression level of CHMP4b along the active infection. Accordingly, CHMP4b expression was not augmented when BMDCs were incubated with heat-shock killed parasites, and importantly, the anti-CHMP4b antibody used does not cross-react with a potential *T. gondii* CHMP4b ortholog (Supplementary Figure S5E).

### U18666A Treatment Inhibits CHMP4b Recruitment to the PV and *T. gondii* Replication

**FIGURE 7:**
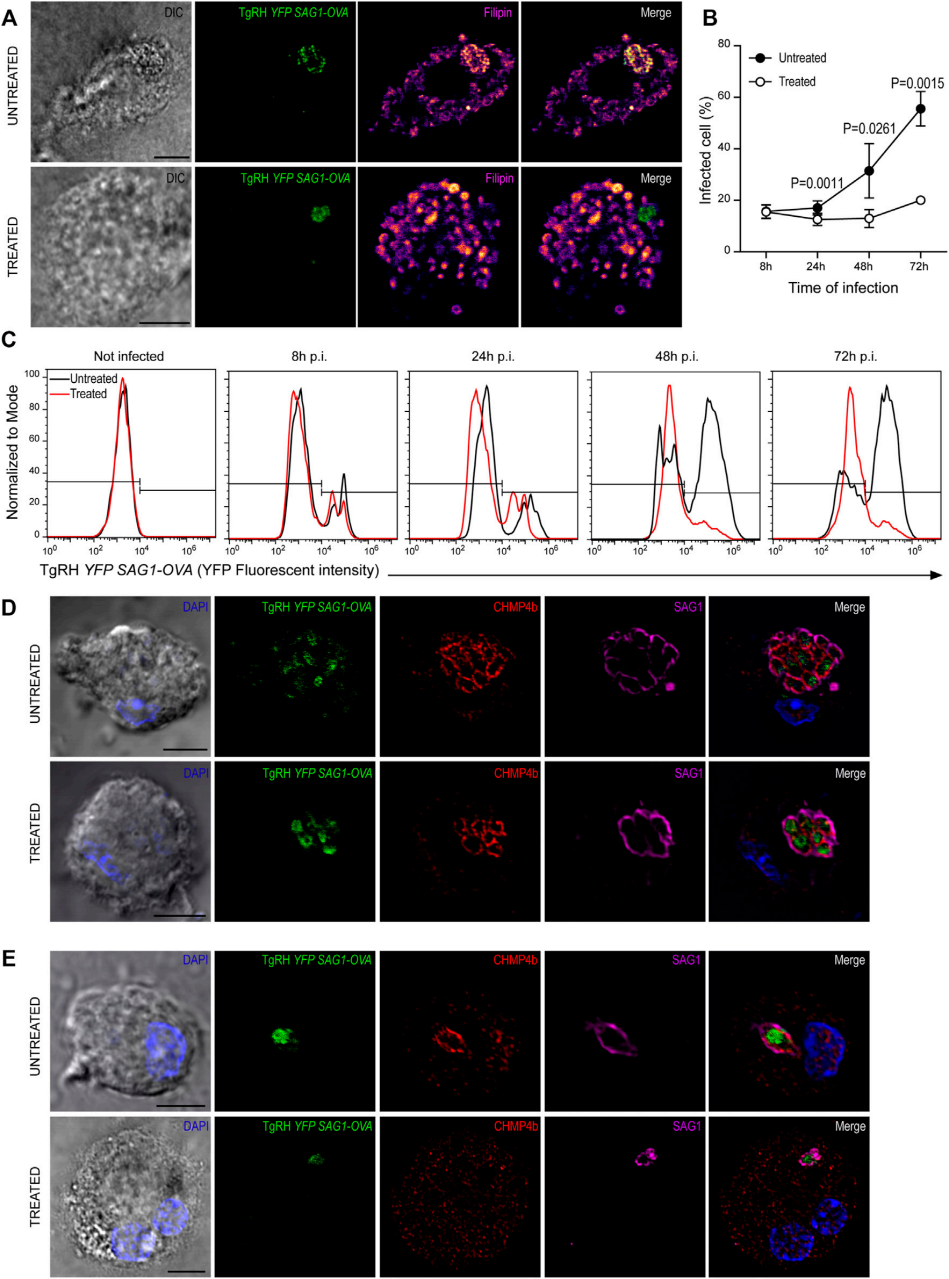
U18666A treatment inhibits *T. gondii* replication. **(A)** Filipin labeling and confocal microscopy analysis showing the distribution of cholesterol in untreated (up) and treated (down) BMDCs infected with TgRH *YFP SAG1-OVA* (green) for 24 h. DIC images are shown on the left and overlay of all fluorescent channels is shown in the right panels. Scale bars: 5 µm. Data are representative of 15 images analyzed for each experimental condition from three independent experiments. **(B)** *T. gondii* proliferation curve in the presence of the inhibitor. Histograms showing the percentage of infected cells. Data represent mean ± SEM of triplicates values from three independent experiments. The *P-*value for each condition is indicted in figure. *p* > 0.05 (ns). The one-tailed Student’s paired *t*-test was performed. **(C)** Representative FACS profile of *T. gondii* fluorescence in untreated (black lines) and treated (red lines) BMDCs. **(D,E)** Immunofluorescence labeling and confocal microscopy analysis showing CHMP4b and SAG1 in untreated and treated BMDCs infected with TgRH *YFP SAG1-OVA* for 48 h. **(D)** Shows big *T. gondii* PVs and **(E)** small *T. gondii* PVs (probably corresponding to a second round of replication). Nuclei stained with DAPI. DIC images are shown on the left, TgRH *YFP SAG1-OVA* (green), CHMP4b (red), SAG1 (magenta), and the overlay of all fluorescent channels is shown in the right panels. Scale bars: 5 µm. Data are representative of 15 images analyzed for each experimental condition from three independent experiments.

Previous work has shown a strong dependence of *T. gondii* replication on host-derived cholesterol supply (Coppens et al., 2000; Bottova et al., 2009; Sanfelice et al., 2017). However, these studies were not carried out in DCs, one of the preferred cellular targets of *T. gondii*. Given the singular interaction between *T. gondii* and DCs, we proposed to evaluate this scenario. For this, BMDCs were treated with 7.5 μg/ml U18666A (or with the equivalent volume of DMSO) during infection with TgRH *YFP SAG1-OVA* at MOI 1, and cholesterol was stained with filipin after 24 h. As shown in Figure 7A, a clear accumulation of cholesterol inside the PV of untreated cells was evidenced. Conversely, in the presence of the inhibitor, cholesterol was accumulated in large granules throughout the host cytosol, but not particularly at the PV.

In order to address if U18666A treatment of BMDCs impacts on *T. gondii* fitness, we followed parasite proliferation by flow cytometry analysis taking advantage of the fluorescence expressed by the TgRH *YFP SAG1-OVA* strain. The fluorescence profiles show that during the first 24 h pi *T. gondii* replicates similarly inside treated and untreated BMDCs. However, a significant delay of fluorescence incremental was observed in the treated condition from 48 h pi onwards, indicating an inhibition of parasite proliferation in these cells (Figure 7C, quantified in Figure 7B). Although a strong recruitment of CHMP4b to the replicating vacuoles was evident for both conditions at 48 h pi, the PVs observed were smaller, in size and number of parasites, in treated BMDCs than those present in untreated cells (Figure 7D). Moreover, CHMP4b was not recruited to small single parasite-containing vacuoles in U18666A-treated BMDCs at 48 h pi, whilst in untreated cells, similar size PVs were strongly decorated with CHMP4b (Figure 7E). To further visualize these differences, the fluorescence intensity of CHMP4b, SAG1 and *TgRH YFP SAG1-OVA* was quantified in multiple and single parasite-containing vacuoles in control and treated cells (Supplementary Figures S6A,B, respectively). This result suggest that after the initial *T. gondii* infection and replication, CHMP4b cannot be efficiently recruited to the PV in successive replication rounds upon BMDC treatment with the drug.

Altogether, our data indicate an important participation of cholesterol in the *T. gondii* – DCs relationship, being necessary for both growth and proliferation, as well as for the optimal transport of MHC-I and MHC-II molecules to the cell surface required for efficient exogenous antigen presentation.

## Discussion

Cholesterol is a highly hydrophobic lipid that requires very tightly regulated molecular mechanisms to be transported along the cell. By and large, the efficacy of intracellular cholesterol transport depends on a proper subcellular distribution among organelles and the plasma membrane. This represents an essential feature of healthy cells that allows them to perform critical functions (Soccio and Breslow, 2004). In this study, we were interested to investigate the relevance of intracellular cholesterol transport during exogenous antigen presentation by DCs, which represent the most potent antigen presenting cells of the immune system. As general strategy, we altered cholesterol transport by the use of the drug U18666A, which has been widely characterized as a powerful inhibitor of NPC1 function and thereby retains cholesterol inside endolysosomes (Lu et al., 2015). We decided to address this study by generating primary cultures of DCs differentiated from bone marrow of C57BL/6 mice. Hence, we set up a working concentration of U18666A that was not toxic for BMDCs, but clearly modified the intracellular distribution of cholesterol, as evidenced by filipin labeling and confocal microscopy. Although this experimental system was convenient to address our study, it also represents a limitation since we did not use other methods to change cholesterol levels or its transport. Besides, pharmacological intervention is not always a very specific approach because it may affect other important intracellular parameters.

A marked defect of MHC-I and MHC-II antigen presentation was evidenced in U18666A-treated BMDCs when we tested soluble, particulate and *T. gondii*-associated antigens. For the case of MHC-II antigen presentation, we also found a significant inhibition of CD4+ T lymphocyte activation by treated DCs when they were incubated with the corresponding short control peptides (OVA_(323–339)_ for OT-IIZ and CD4Ag28m_(605–619)_ for BTg01Z cells). However, the activation of B3Z CD8+ T cells upon BMDC incubation with the short control peptide OVA_(257–264)_ was not diminished in treated cells. Accordingly, the deficiency of MHC-II molecule transport to the cell surface in treated BMDCs was stronger than for MHC-I, as analyzed by flow cytometry. A lack of flaw in the skill of U18666A-treated BMDCs to internalize exogenous antigens, make us consider that the inhibition of MHC-I and MHC-II transport to the cell surface is responsible, at least in part, of the defective antigen presentation phenotype that these cells exhibit. This conclusion is supported by the observation that the presentation defect observed in BMDCs from NPC1^−/−^ mice is corrected when a subpopulation of cells with normal levels of MHC-II at the plasma membrane is selected (Bosch et al., 2013a).

In line with our results, previous studies have reported a loss of MHC-II expression at the cell surface when cholesterol transport is hampered (Kuipers et al., 2005; Vrljic et al., 2005) or a stability inhibition of MHC-II/peptide complexes accumulated at the plasma membrane in microclusters when cholesterol is depleted (Bosch et al., 2013b; Roy et al., 2013). An interesting experimental setup involving *Leishmania* infected macrophages demonstrate the relevance of cholesterol supply for efficient MHC-II antigen presentation (Roy et al., 2016). Regarding MHC-I expression, there is not much information available about the potential effect of cholesterol depletion, or cholesterol transport inhibition, during MHC-I trafficking to the cell surface. However, it was shown that cholesterol depletion with lovastatin impairs macropinocytosis and consequently, the MHC-I cross-presentation capacity of treated DCs was reduced (Albrecht et al., 2006). The use of U18666A in our experiments interrupts cholesterol transport without affecting fluid-phase endocytosis. Therefore, our study brings novel data about the relevance of cholesterol trafficking during MHC-I cross-presentation independently of the exogenous antigen uptake.

The striking defect observed for both MHC-I and MHC-II presentation of *T. gondii*-associated antigens, motivated us to investigate deeper into other aspects of the parasite fitness. The protozoan *T. gondii* is an obligate intracellular parasite that cannot synthesize sterols and must scavenge lipids from the host cell in order to grow and replicate inside the PV. This lipid acquisition includes cholesterol endocytosis by the parasite via the lysosomal low-density lipoprotein pathway (Coppens et al., 2000). Moreover, *T. gondii* also sequesters LDs, which represent a major intracellular source of cholesterol, and internalizes these organelles into the vacuole to achieve efficient proliferation (Hu et al., 2017; Nolan et al., 2017). In summary, different publications highlight the importance of host cholesterol supply for optimal *T. gondii* replication (Bottova et al., 2009; Sanfelice et al., 2017). In this sense, we provide further evidence to support this concept by showing that U18666A treatment of BMDCs inhibited cholesterol recruitment to the PV. As consequence, correct PV development and parasite growth were strongly blocked in our system. More importantly, our study is pioneer in addressing the relevance of cholesterol transport during *T. gondii* antigen presentation since this had not been tested before.

Probably one of the most remarkable findings of this study is the massive recruitment of CHMP4b to the PV and the subsequent over-expression of this ESCRT-III member in BMDCs infected by *T. gondii*. In accordance with our results, a very recent study shows that *T. gondii* drives the recruitment of the host ESCRT machinery through the parasite effector transmembrane dense granule protein TgGRA14. The interaction between TgGRA14 and host ESCRT components allows the efficient uptake of cytosolic proteins from the host cell important for parasite survival (Rivera-Cuevas et al., 2021). Another recent work from a different group that used a proximity-labeling strategy in combination with quantitative proteomics demonstrated that three components of the host ESCRT machinery are present at the host-parasite membrane interface in infected fibroblasts (Cygan et al., 2021). Interestingly, the vacuolar space of *T. gondii* PV comprises a sophisticated membranous IVN formed of tubules and vesicles. In this study, we also found round intraluminal vesicles in the vacuolar space of the parasite by performing both TEM and FIB-SEM experiments. The question of how these vesicles are generated inside the PV and if the host ESCRT machinery plays a relevant role in this process during the recruitment to the *T. gondii* vacuole remains open. Ongoing and future studies involving CHMP4b silencing in DCs will allow us to formally test this idea and define a more precise role for the ESCRT machinery during *T. gondii* infection.

## Data Availability

The raw data supporting the conclusions of this article will be made available by the authors, without undue reservation.

## Supplementary Material

Supplementary Figure 1 U18666A treatment does not affect the viability of BMDCs. **(A)** Getting strategy used to determine the cell population to analyze. **(B)** Representative FACS profiles of ToPro3 fluorescent intensity corresponding to apoptotic cells. **(C)** Percentage of ToPro3 negative cells in control condition (black bar) and treated condition (white bar), after 24 h of treatment.

Supplementary Figure 2 MHC-I and MHC-II molecules are accumulated in different intracellular endocytic compartments upon U18666A treatment. Immunofluorescence labeling and confocal microscopy analysis of U18666A-treated and untreated BMDCs showing **(A)** H-2K^b^ MHC-I (green) and Rab11a (red), and **(B)** filipin (green) and I-A^b^ MHC-II (red). Nuclei stained with DAPI. DIC images are shown on the left and the overlay of all fluorescent channels is shown in the right panels. Scale bars: 5 µm. Data are representative of 10 images analyzed for each labeling.

Supplementary Figure 3 MHC-I and MHC-II molecules expression by FACS analysis. Representative FACS profiles of untreated and U18666A-treated BMDCs in intact (surface staining) and permeabilized (intracellular staining) cells showing the fluorescence intensity of H-2K^b^-FITC (upper panels) and I-A^b^-PE(lower panels) antibody labeling.

Supplementary Figure 4 3D imaging of an entire PV by FIB-SEM. HFF infected for 24 h with TgRH *YFP SAG1-OVA* were fixed and prepared for FIB-SEM imaging. This movie shows a stack of 1731 images binned in xy and cropped around the vacuolar space that contains four tachyzoites. Voxel size is 10 × 10 × 5 nm (xyz). Movie related to Figure 5B which shows an excerpt of three slices from the stack: #347, #519, and #852.

Supplementary Figure 5 MVBs markers distribution in not infected BMDCs. Immunofluorescence labeling and confocal microscopy analysis of non-infected BMDCs showing **(A)** CD63, **(B)** TSG101 and **(C)** CHMP4b in red, and **(D)** BMDCs infected with TgRH *YFP SAG1-OVA* for 48 h showing the parasites in green, CHMP4b in red and SAG1 in magenta. Nuclei stained with DAPI. DIC images are shown on the left and the overlay of all fluorescent channels is shown in the right panels. Scale bars: 5 µm. Data are representative of 10 images analyzed for each labeling. **(E)** Immunoblotting of CHMP4b and SAG1 in BMDCs infected for 48 h with TgRH *YFP SAG1-OVA* at MOI 6 (1); in BMDCs that phagocytosed the heat killed and opsonized *T. gondii* strain TgRH *YFP SAG1-OVA* during 24 h (2) or 48 h (3), and in total lysates of the *T. gondii* strains TgRH *YFP SAG1-OVA* (4) or TgPru *Tomato SAG1-OVA* (5).

Supplementary Figure 6 Fluorescence intensity profiles of CHMP4b in *T. gondii* PVs. Immunofluorescence labeling and confocal microscopy analysis related to Figures 7D,E showing the fluorescence intensity profiles of TgRH *YFP SAG1-OVA* (green), CHMP4b (red) and SAG1 (magenta) in **(A)** multiple and **(B)** single parasite-containing vacuoles of infected BMDCs. Straight lines were drawn delimiting the analyzed area.
