# Supplementary figures and images for: Efficient Cholesterol Transport in Dendritic Cells Defines Optimal Exogenous Antigen Presentation and Toxoplasma gondii Proliferation
Source: Front Cell Dev Biol. 2022 Mar 4;10:837574. doi: 10.3389/fcell.2022.837574 (PMC8931308; doi:10.3389/fcell.2022.837574)

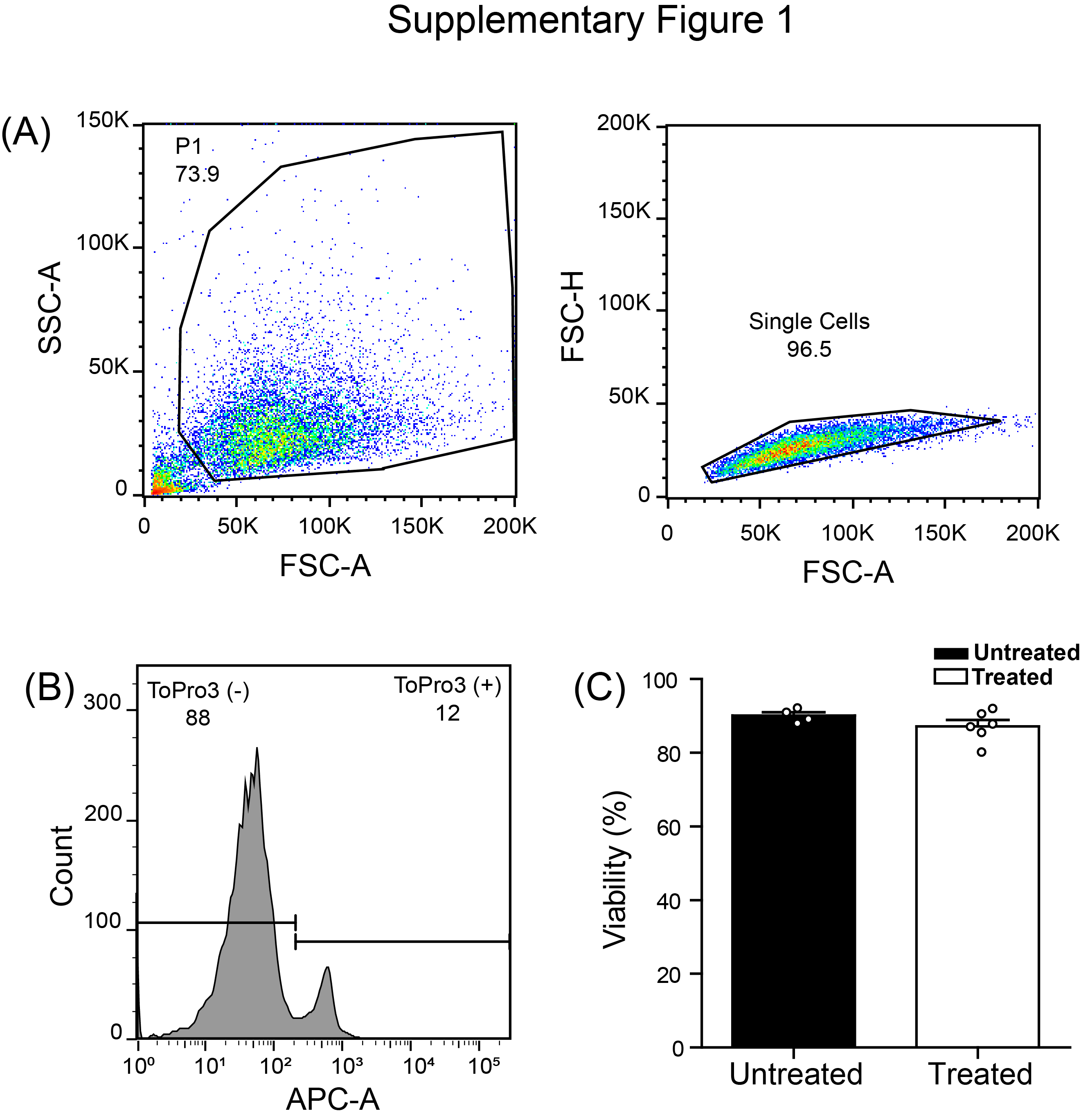

Supplement: Supplementary file 1 [file Presentation1.zip › Fig. S1.tif]

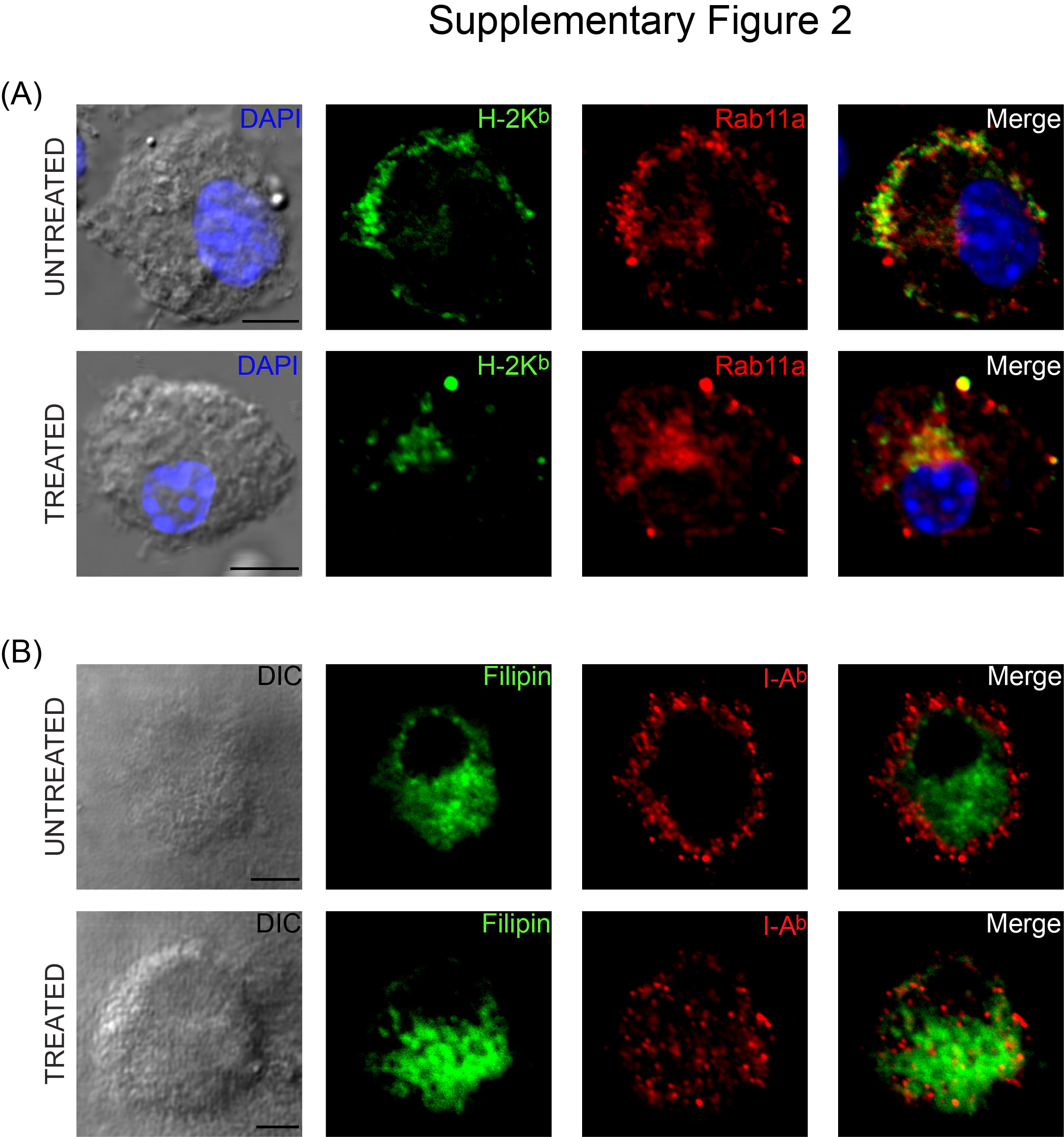

Supplement: Supplementary file 1 [file Presentation1.zip › Fig. S2.tif]

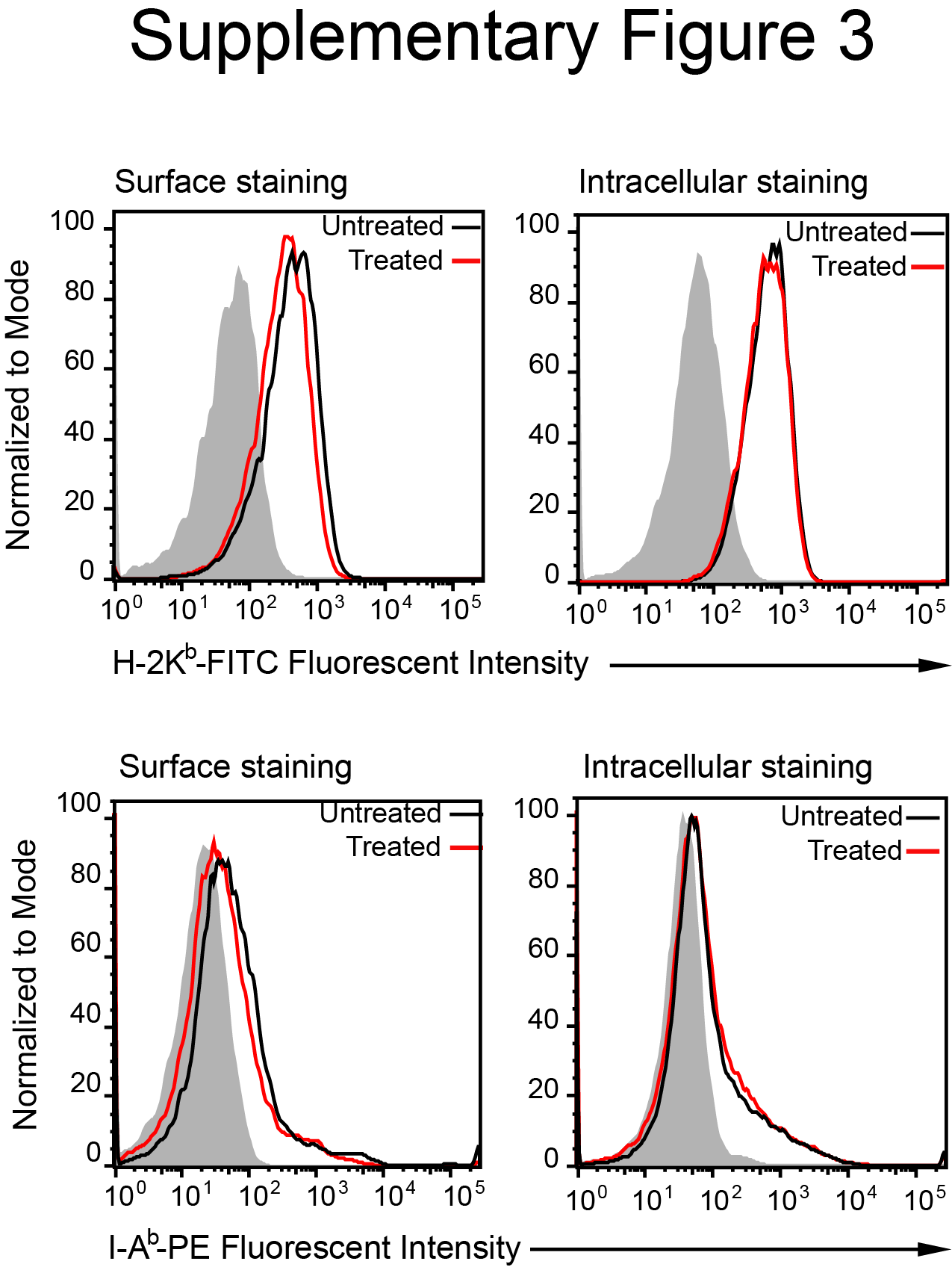

Supplement: Supplementary file 1 [file Presentation1.zip › Fig. S3.tif]

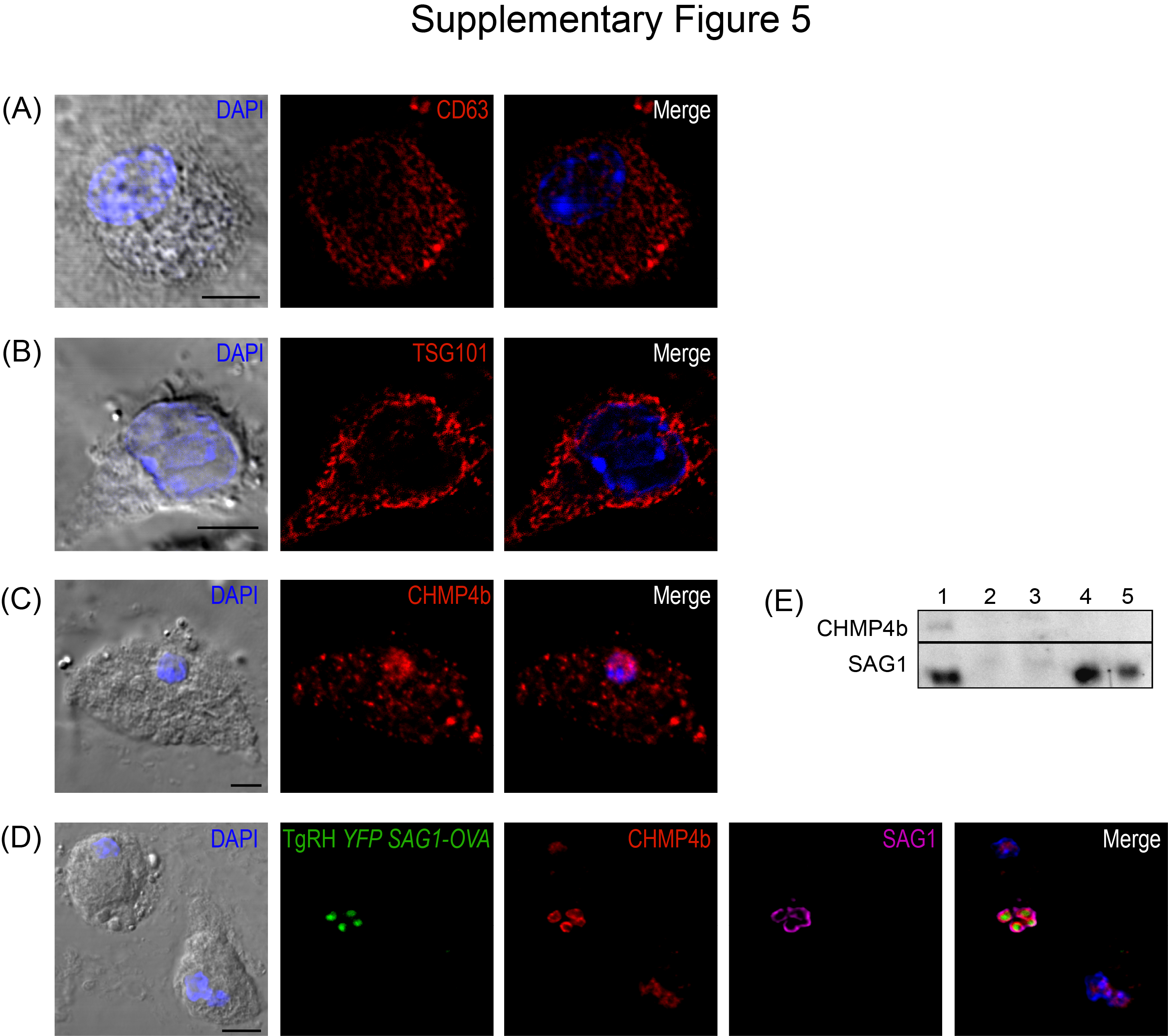

Supplement: Supplementary file 1 [file Presentation1.zip › Fig. S5.tif]

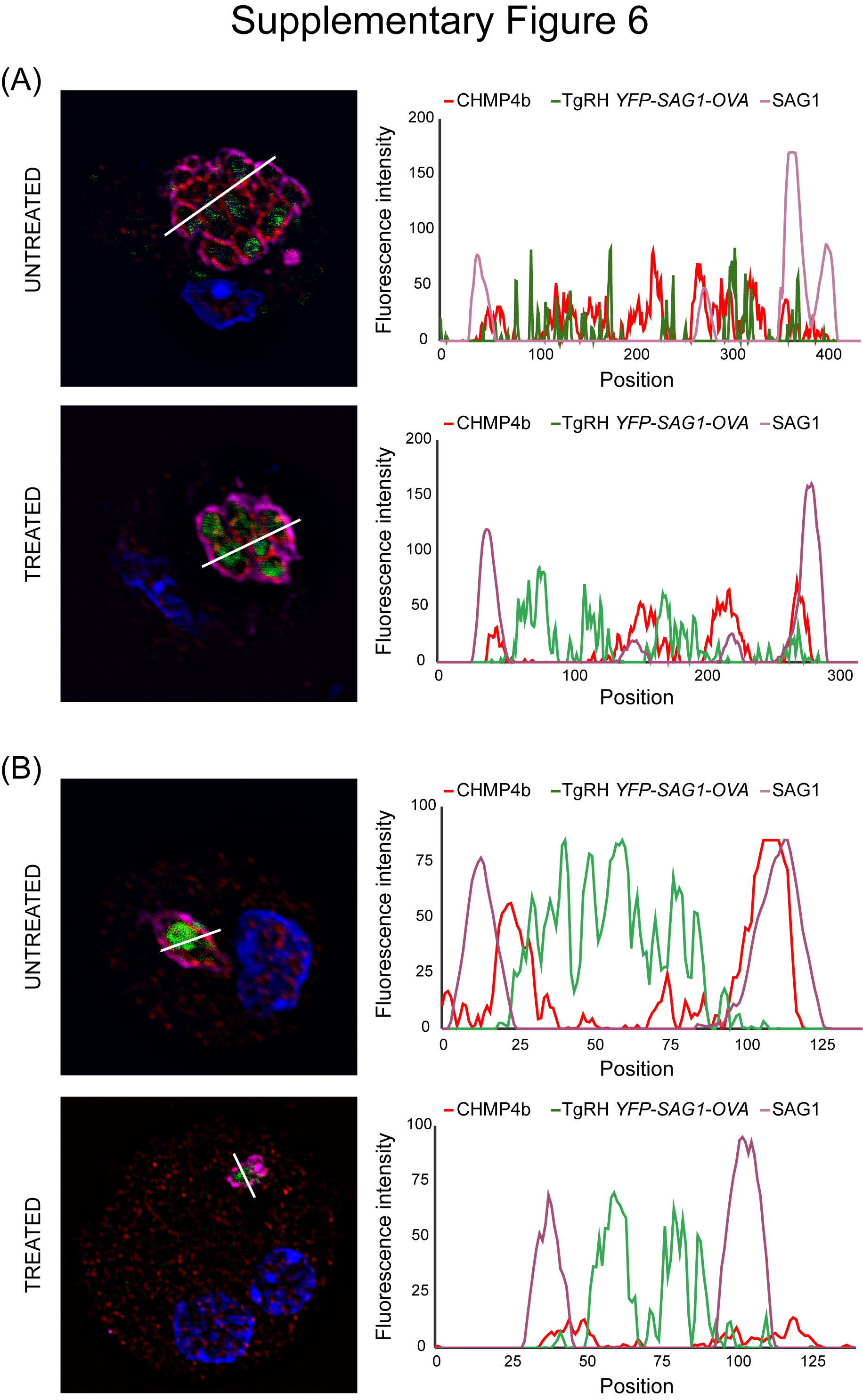

Supplement: Supplementary file 1 [file Presentation1.zip › Fig. S6.tif]
